# Hepatitis B and C Viruses Infections and Their Association with Human Immunodeficiency Virus: A Cross-Sectional Study among Blood Donors in Ethiopia

**DOI:** 10.4314/ejhs.v21i1.69047

**Published:** 2011-03

**Authors:** Alemeshet Yami, Fissehaye Alemseged, Alima Hassen

**Affiliations:** 1 Department of Internal Medicine, Jimma University; 2 Department of Epidemiology, Jimma University; 3 Department of Pharmacy, Jimma University

**Keywords:** Hepatitis B, Hepatitis C, HIV, blood donors, Southwest Ethiopia

## Abstract

**Background:**

Since the introduction of Highly Active Anti-Retroviral Therapy and the dramatic improvement in the prognosis of individuals with Human Immunodeficiency Virus, liver disease due to chronic viral hepatitis has become as important cause of morbidity and mortality in co-infected individuals. The objective of the study was to determine the Sero-prevalence of Hepatitis B Virus, Hepatitis C Virus and Human Immunodeficiency Virus and the association of the virus with Hepatitis B Virus and Hepatitis C Virus infection. As Human Immunodeficiency Virus and Hepatitis B Virus infections are highly prevalent and they are among the major public health concern in developing countries including Ethiopia investigating this problem is of paramount benefit. Although studies on co-infection of Hepatitis C Virus and Human Immunodeficiency Virus have clearly identified adverse effects of co-infection, the prevalence of Hepatitis C Virus infection and the association with Human Immunodeficiency Virus in developing countries including Ethiopia has not been know for sure.

**Method:**

A cross sectional study was conducted from January 1 to 31, 2010, in Jimma University specialized hospital Blood Bank. The inclusion criteria of the study was adult who donated blood to Jimma University specialized hospital blood bank any time from establishment of the unit until January 2010 and whose record was retrieved. Accordingly 9,204 adults were included of which 6,063 were selected by lottery method. Data on socio-demographic variables (age and sex), laboratory test result for Hepatitis B surface Antigen, anti-Hepatitis C Virus antibody, anti-Human Immunodeficiency Virus 1 antibody, and Rapid Plasma Reagin tests were collected using structured questionnaire. After the data were collected, they were entered into a computer and analyzed using SPSS -16 for windows. P-Value of < 0.05 was taken to be statistically significant.

**Results:**

The prevalence rate of Hepatitis B Virus, Hepatitis C Virus, Human Immunodeficiency Virus and syphilis infection were 2.1%, 0.2%, 2.1% and 0.7%, respectively. Sex and age had statistically significant association with Human Immunodeficiency and Hepatitis B virus infections where females were less likely to be infected. As age increases above 20 years, the risk of infection with Human Immunodeficiency Virus or Hepatitis B Virus increases. There was no association between Hepatitis B Virus, Hepatitis C Virus and Human Immunodeficiency Virus.

**Conclusion:**

the prevalence rate of Hepatitis B Virus, Hepatitis C Virus and Human Immunodeficiency Virus infections among blood donors in Jimma University specialized hospital were lower as compared to previous studies, in addition there was no association between Hepatitis B Virus, Hepatitis C Virus and Human Immunodeficiency Virus. Thus, community based study should be conducted to confirm the relationship of Hepatitis B Virus, Hepatitis C Virus and Human Immunodeficiency Virus.

## Introduction

Since the introduction of HAART and the dramatic improvement in the prognosis of individuals with HIV, liver disease due to chronic viral hepatitis has become an important cause of morbidity and mortality in co-infected individuals (1). This fact brought a serious concern about the growing problems of HIV/AIDS pandemic in sub Saharan Africa; where the transmission is predominantly through sexual route and most of the people in these region are already been exposed to HBV by the time they become sexually active to acquire HIV infection, with minority being exposed to both viruses more or less simultaneously (2, 3). Evidences indicate that HIV positive individuals are more likely to be infected with HBV, to be chronic carrier and have a higher HBV replication rate than HIV negative individuals. In addition, it is evident that immuno-suppression brought about by HIV infection may cause re-activation or re-infection in those previously exposed to HBV; further more HIV infection exacerbates liver disease in HBV co-infected individuals and there is an even greater risk of liver disease when HIV and HBV co-infected patients are treated with HAART (3).

Most of the researches done on HIV and HBV co-infection have been conducted in areas of low HBV endemic regions where a number of studies carry similar ideas; a significant higher prevalence of HBV infection in HIV positive individuals. Studies done in sub-Saharan Africa have conflicting result; most of which don't support an increase prevalence of HBV in HIV positive individuals (3, 4–9) and very few studies about HBV-HIV co-infection exist in Ethiopia. A case in point is a study done on male blood donors appearing at the blood bank of a regional hospital in Northwest Ethiopia where the seroprevalence of HBsAG was 14.4% and the prevalence of HIV infection was 16.7% and there was no association between HIV and HBV infection(OR=0.79,95% CI=0.361.67) (10). Another study done in Addis Ababa found that: the prevalence of HBsAg and anti-HBc in VCT clients was 5.7% and 44.8%, respectively. Among HIV-infected persons, 3.9% were sero-positive for HBsAg; and there was no significant difference in HBsAg or anti-HBc sero-positivity between HIV-positive and HIV-negative subjects. Anti-HBc positivity was significantly higher in men, among the age range 40–49 years, and in subjects with a history of catheterization (11). A comparative cross-sectional study in asymptomatic Ethiopian compared data on the 52 people who were HIV positive with 139 who were HIV negative to examine HIV's relationships with treponemal infection and hepatitis B infection. The two groups were essentially the same age (37 years for cases and 35.5 years for controls). No significant difference in the prevalence of hepatitis B virus (HBV) markers existed between the two groups (70% for HIV-positive migrants and 78.8% for HIV-negative migrants), but HIV-positive migrants were more likely to have markers for hepatitis B surface antigen than HIV-negative migrants (20% vs. 8.6%; p=0.018). The HIV-positive migrants had a higher prevalence of treponemal markers than did HIV-negative migrants (31% vs. 3%), this finding was interpreted as indicating that treponemal disease increased their risk of HIV infection (12).

HIV and HCV share common mechanism of transmission (parental, sexual and mother to child). This epidemiological similarity accounts for the high frequency of co-infection by HIV and HCV, which is common among patients with history of injectable drug use and blood transfusion (13, 14). Co-infection of HCV in HIV–infected patients have several implications for clinical care and management; firstly, HCV viral load is higher in people with HIV infection, the rate of progression of Hepatitis C infection to end stage liver disease is accelerated 3 fold in the presence of HIV infection and HCV infection is now a leading cause of hospitalization and death in this population (15, 16). Secondly hepatitis C increases the risk of hepato-toxicity with ART, particularly with drugs such as nevirapine, and so may affect the choice of therapy and timing (15, 17). Thirdly, the use of HAART in HCV infected patients may precipitate HCV immune reconstitution phenomena, and there may also be impaired immune recovery in co-infected individuals (18, 19). A multi center study done to assess HCV prevalence and predictors of HIV/ HCV co-infection has found that: HIV/HCV co-infection prevalence was 16.1%, which varied from 1.9% in South Africa to 48.6% in Italy. The strongest predictors of HIV/HCV co-infection was HIV exposure category (P< 0.001), the prevalence of HIV/HCV was low (3.7%) among homosexual men without reported IDU; this may suggest the low efficacy of sexual transmission of HCV (20). Few studies about HCV-HIV co-infection exist in sub-Saharan Africa. A study done in Kenya to determine the prevalence of HCV and HCV/HIV co-infection among medical inpatients found similar prevalence of HCV in both HIV positive and negative patients (3.7% Vs 4.4 % respectively) (21). Another study done in Ethiopia to determine the prevalence of HCV in antenatal attendees and commercial sex workers found that the overall prevalence of HCV was 1.8% peaking to 5.6% in 40–44 years old age group and HIV prevalence was 4.9%. Yet, sero-prevalence of HCV was markedly increased in HIV infected sera that HIV negative samples (8.6% Vs 1.6%)(36). A study in Addis Ababa found that HCV antibody prevalence was 0.9% and higher among HIV-positive compared to HIV-negative individuals (4.5% vs. 0.8%, respectively, P < 0.001). Similar higher prevalence of HCV antibodies was seen among HIV-positive compared to HIV-negative antenatal care attendees (2.9% vs. 0.8%, respectively, P=0.003), and sex workers (5.3% vs. 1.3%, respectively, P=0.02). After stratification by HIV status, HCV prevalence among women of the general population was identical to that of sex workers, suggesting that HCV sexual transmission is not common in this population and that HIV infection does not enhance susceptibility to HCV sexual transmission (22).

As Human Immunodeficiency Virus and Hepatitis B Virus infections are highly prevalent and they are among the major public health concern in developing countries including Ethiopia, investigating this problem is of paramount benefit. Although studies on co-infection of Hepatitis C Virus and Human Immunodeficiency Virus have clearly identified adverse effects of co-infection, the prevalence of Hepatitis C Virus infection and the association with Human Immunodeficiency Virus in developing countries including Ethiopia has not been know for sure. This study was conducted in JUSH Blood Bank Unit to determine the sero-prevalence of HBV, HCV and HIV and ascertain association of HIV infection with HBV and HCV infections in Ethiopia. Jimma University specialized Hospital (JUSH) is the only hospital in Jimma zone serving the majority of people living in Jimma town and its surrounding. JUSH provides both inpatient and outpatient services and as one of the inpatient services the hospital has blood bank unit. The Blood bank unit is established in 1987 and started functioning since 1988. The unit is currently run by Jimma University specialized hospital in collaboration with Jimma Red Cross Society. Since the establishment of the unit, about 600 to 900 adults donate blood yearly.

## Methods and Materials

A cross-sectional study was conducted from January 1 to 31, 2010 in JUSH Blood Bank Unit. The unit receives and stores blood donated from adults either as replacement or voluntarily. Regular screening of blood donors is made before donation by in-depth interview for symptoms of disease and by measuring weight, blood pressure and hemoglobin level. Those adults who are apparently asymptomatic, weighing 50 Kg or more, with normal blood pressure and hemoglobin level donate blood. After collection of donated blood, samples are taken for screening of HIV, HBV and HCV infections using ELISA technique for anti HIV-1 antibody, HBsAG, and anti HCV-antibody respectively and Rapid Plasma Reagin (RPR) test for syphilis infection. If negative for all four serological tests mentioned above, the blood is stored in refrigerator and delivered for patient that needs blood transfusion. In the Unit every blood sample is given a unique code and recorded. Reports are also delivered to the hospital statistics office and finally to national level every month, quarterly, and annually.

The inclusion criterion was blood donated by an adult to the JUSH blood bank since the establishment of the unit until January 2010 and where the record could be retrieved. Accordingly 9,204 adults were included of which 6,063 were selected by lottery method. Data on socio-demographic variables (age and sex), laboratory test result for HBsAG, Anti-HCV antibody, HIV, and RPR were collected using structured questionnaire. After collection, the data was cleaned manually, categorized and coded finally entered in to a computer and analyzed using SPSS version 16.0 for windows. First, Univariate analysis was done to determine the frequency of positive serological test, then bivariate analysis was done to determine predictors for infection and finally multivariate analysis was done to determine independent predictors of infection using logistic regression model. P-Value < 0.05 was taken to be statistically significant.

Ethical clearance was obtained from Ethical Committee of Jimma University. To avoid the risk of back retrieval of confidential information like names of the donors, during data collection new code numbers were given by the principal investigator. After the necessary information was obtained, the code numbers on the questionnaires were deleted.

## Results

Of the total 6063 blood donors, males were 4802 (79.2%) and 1261 (20.8%) were females. Their mean age was 26.7±8.6 years (range 18–67). The majority (91.6%) of donors were < 45 years old and majority of them, 4102 (67.7%) donated blood from September 2008 to January 2010 (Table 1).

**Table 1:** Base line characteristics of blood donors in JUSH, January 2010.

	Variables	Number (%)
Sex	Male	4802 (79.2)
	Female	1261 (20.8)
Age	<20	1472 (24.3)
	20–24	1856 (30.6)
	25–29	957 (15.8)
	30–34	957 (15.8)
	35–39	509 (8.4)
	40–44	509 (8.4)
	≥ 45	509 (8.4)
Year of donation	Before Sep. 2008	1961 (32.3)
	Sep.2008–Jan.2010	4102 (67.7)

Total		6063 (100.0)

The prevalence rate of HBV, HCV, HIV and syphilis infection as evidence with positive serological test for HBsAG, anti HCV antibody, anti HIV-1 antibody and RPR were 126 (2.1%), 10 (0.2%), 129(2.1%), and 44(0.7%), respectively (Table 2).

**Table 2:** Serological test result of blood donors in JUSH, January 2010.

Variables	Status	Number (%)
HBsAG	Positive	126(2.1)
	Negative	5937(97.9)

HIV	Positive	129(2.1)
	Negative	5934(97.9)

Anti –HCV antibody	Positive	10(0.2)
	Negative	6053(99.8)

RPR	Positive	44(0.7)
	Negative	6019(99.3)

Total		6063(100.0)

The overall prevalence rate of HIV infection in the study population was 2.1%. One hundred nineteen (2.5%) out of the 4802 males and 10 (0.8%) out of the 1261 females were having HIV infection and analysis of HIV infection by age group revealed prevalence increases as age increases. The prevalence rate of HIV infection was higher in those who donated blood before September 2008 as compared to those who donated later; 47 of 1961 (2.4%) and 82 of 4102(2.0%) respectively. The prevalence rate of HIV was lower in those having HBV infection as compared to those without the infection; 2 of 126 (1.6%) and 127 of 5937 (2.1%) respectively. On the other hand, none of the HIV positive blood donors had co-infection with HCV or syphilis. Furthermore, Bivariate analysis showed association of HIV infection with Sex and age, this association was also maintained on multivariate analysis. Accordingly, females were less likely to be infected with HIV as compared to males, adjusted OR = 0.381, 95%CI, (0.198, 0.733). As compared to those in the age group less than 20 years, those of age 20–29 years, 30–34 years, 35–39 years and 40–44 years were two, four, five and eight times at increased risk of HIV infection ,respectively. There was no statistically significant difference in the prevalence of HIV infection among the age group less 20 years and those with age group of 45 years or more; adjusted OR = 0.315, 95%CI (0.040, 2.450). Furthermore, there was no association among year of donation, HCV infection, HBV infection and Syphilis with HIV infection (Table 3).

**Table 3:** HIV infection in of blood donors in JUSH, January 2010.

Variables		Number (%)	HIV positive Number (%)	P-value	Crude OR (95%ci)	Adjusted OR (95%ci)
Sex	Male	4802(79.2)	119(2.5)	0.000	1	
	Female	1261(20.8)	10(0.8)		0.315(0.164, 0.602)	0.381(0.198,0.733)

Age	<20	1472 (24.3)	11(0.7)	0.000	1	
	20–24	1856(30.6)	33(1.8)		2.404(1.211, 4.774)	2.162(1.086, 4.302)
	25–29	957(15.8)	18(1.9)		2.546(1.197, 5.415)	2.193(1.027,4.681)
	30–34	957(15.8)	23(3.7)		5.049(2.446,10.423)	4.329(2.088, 8.973)
	35–39	509(8.4)	23(4.5)		6.286(3.042,12.988)	5.364(2.585,11.133)
	40–44	509(8.4)	20(6.8)		9.766(4.627, 20.613)	8.491(4.008,17.988)
	≥ 45	509(8.4)	1(0.3)		0.382(0.049,2.966)	0.315(0.040,2.450)

Year of donation	Before Sep. 2008	1961(32.3)	47(2.4)	0.315		
	Sep.2008–Jan.2010	4102(67.7)	82(2.0)			

Anti HCV antibody	Positive	10(0.2)	0(0.0)	0.625		
	Negative	6053(99.8)	129(2.1)			

HBsAG	Positive	126(2.1)	2(1.6)	0.671		
	Negative	5937(97.9)	127(2.1)			

RPR	Positive	44(0.7)	0(0.0)	0.326		
	Negative	6019(99.3)	129(2.1)			

Total		6063(100.0)	129(2.1)			

The overall prevalence rate of HBV infection in the study population was 2.1% where 119 (2.5%) out of the 4802 of the males and seven (0.6%) of the 1261 females were having HBV infection. Analysis of HBV infection by age group showed highest prevalence in the age group 40–44 years and lowest among the age group of forty five years or more 13 of 509 (4.5%) and 3 of 509 (0.9%) respectively. The prevalence rate of HBV infection was similar, 2.1% in those who donated blood before September 2008 and in those who donated later. However, the prevalence rate of HBV is low in those having HIV infection as compared to those without the infection and only two of 129 (1.6%) of blood donors with HIV infection and 124 (2.4%) of blood donors without HIV infection were positive for HBV but the prevalence rate of HBV was higher in those having Syphilis infection as compared to those without it where two (4.5%) of the 44 blood donors with Syphilis infection and 124 (2.1%) of 6019 of blood donors without Syphilis infection were positive for HBV; yet, none of HBV positive blood donors had co-infection with HCV. Furthermore, Bivariate analysis revealed that sex and age had association with HBV infection where this had also been maintained on multivariate analysis. On the other hand, females were less likely to be infected with HBV as compared to males, adjusted OR = 0.243, 95%CI (0.112, 0.524). As compared to those in the age group less than 20 years, those with age group 20–24 years, 30–34 years and 35–39 years were twice and those with age group 40–44 years were four times at increased risk of HBV infection, respectively. But there was no association between year of donation, HCV infection, HIV infection and syphilis with HBV infection (Table 4).

**Table 4:** HBV infection in blood donors in JUSH, January 2010.

Variables		Number (%)	Pos. for HBsAG Number (%)	P-value	Crude OR (95%CI)	Adjusted OR (95%CI)
Sex	Male	4802(79.2)	119(2.5)	0.000	1	
	Female	1261(20.8)	7(0.6)		0.220(0.102,0.472)	0.243(0.112, 0.524)

Age	<20	1472 (24.3)	14(1.0)	0.000	1	
	20–24	1856(30.6)	48(2.6)		2.765(1.518, 5.034)	2.413(1.322, 4.404)
	25–29	957(15.8)	16(1.7)		1.771(0.860, 3.645)	1.462(0.708, 3.019)
	30–34	957(15.8)	18(2.9)		3.073(1.519, 6.218)	2.522(1.242, 5.121)
	35–39	509(8.4)	14(2.8)		2.945(1.394, 6.222)	2.402(1.133, 5.092)
	40–44	509(8.4)	13(4.5)		4.853(2.256,10.435)	4.049(1.877, 8.737)
	>_45	509(8.4)	3(0.9)		0.903(0.258,3.159)	0.706(0.201, 2.476)

Year of donation	Before Sep.2008	1961(32.3)	41(2.1)	0.962		
	Sep. 2008–Jan.2010	4102(67.7)	85(2.1)			

HIV	Positive	129(2.1)	2(1.6)	0.671		
	Negative	5934(97.9)	124(2.1)			

Anti-HCV antibody	Positive	10(0.2)	0(0.0)	0.629		
	Negative	6053(99.8)	126(2.1)			

RPR	Positive	44(0.7)	2(4.5)	0.250		
	Negative	6019(99.3)	124(2.1)			

Total		6063(100.0)	126(2.1)			

The overall prevalence rate of HCV infection in the study population was 0.2% where nine (0.2%) of the 4802 males and 2 (0.2%) of the 1261 females were having HCV infection. Analysis of HCV infection by age group showed no evidence of infection among the age group less than 20 years, 20–24 years and in those 45 years or more, but higher prevalence was observed among the age group 30–34 years and 35–39 years, 3 of 957 (0.5%) and 4 of 509 (0.8%), respectively. The prevalence rate of HCV infection was similar in those who donated blood before September 2008 as compared to those who donated later 2 of 1961 (0.1%) and 9 of 4102(0.2%), respectively. The prevalence rate of HCV is higher in those having syphilis infection as compared to those without the infection; one of 44 (2.3%) of blood donors with syphilis infection and 10 of 6019 (0.2 %) of blood donors without syphilis infection were positive for HCV but none of HCV positive blood donors had co-infection with HBV or HIV. Bivariate analysis showed association between syphilis and HCV infection, accordingly blood donors without serological evidence of syphilis infection were at lesser risk of having HCV infection as compared to those having serological evidence of syphilis infection, crude OR = 0.072, 95%CI (0.009, 0.571). However, this association was not maintained on multivariate (Table 5).

**Table 5:** HCV infection of blood donors in JUSH, January 2010.

Variables		Number (%)	Post. for Anti HCV anti-body Number (%)	P-value	Crude OR (95%CI)	Adjusted OR (95%CI)
Sex	Male	4802(79.2)	9(0.2)	0.831		
	Female	1261(20.8)	2(0.2)			

Age	<20	1472 (24.3)	0(0.0)	0.951		
	20–24	1856(30.6)	0(0.0)			
	25–29	957(15.8)	3(0.3)			
	30–34	957(15.8)	3(0.5)			
	35–39	509(8.4)	4(0.8)			
	40–44	509(8.4)	1(0.3)			
	>_45	509(8.4)	0(0.0)			

Year of donation	Before Sep. 2008	1961(32.3)	2(0.1)	0.315		
	Sep.2008–Jan.2010	4102(67.7)	9(0.2)			

HBsAG	Positive	126(2.1)	0.(0.0)	0629		
	Negative	5937(97.9)	11(0.2)			

HIV	Positive	129(2.1)	0(0.0)	0.625		
	Negative	5934(97.9)	11(0.2)			

RPR	Positive	44(0.7)	1(2.3)	0.013	1	1
	Negative	6019(99.3)	10(0.2)		0.072(0.009, 0.571)	0.140(0.017,1.162)

Total		6063(100.0)	10(0.2)			

## Discussion

This study is the first in its kind in Ethiopia as it tried to ascertain association of HIV infection with HBV and HCV infections and was conducted in a large number of population; but as the study population are highly selected group; blood donors, conclusion might not be drawn to general population. The prevalence of HIV (2.1%) is lower than what had been reported by studies done on male blood donors (16.7%) (10), urban setting (5.5%) but higher than rural setting (0.7%) (23), the result is comparable to the prevalence among women attending antenatal clinics which was 1.9% in 2000, 2.6% in 2003 and 2.2% in 2005 (24). There was association between sex and HIV infection where women were less likely to be infected as compared to men; this is against the previous study which showed prevalence among adult women double that of men (23) this could be because the different study population used in the two studies (blood donors versus general population). This study revealed that as age increases above 20 years, the risk of acquiring HIV increases but not for those 45 years or more, this might be because as age increases the risk of exposure to HIV increases, the absence of association in those more than 45 years could be because of the smaller number of individual in this age group, only 509 were in the age group as compared to in the age group 20–24 years, 1856. The study showed no association between HIV with HBV and HCV infections, this is in accordance to previous studies (10, 11, 21).

The prevalence rate of HBV infection using ELISA for HBsAG was 2.1%, this is lower than previous study done on male blood donors which showed prevalence rate of 14.4%(10) and VCT clients that showed the prevalence rate of 5.7%(11) this might be because the study population are highly selected, most of them could be volunteer blood donors and confident of their sero status. Multivariate analysis showed that association between sex and HBV infection exist, where females had lesser risk of infection as compared to males which is in accordance with previous study finding (11). This study revealed that as age increases above 20 years, the risk of acquiring of HBV increases as except for those age 45 years or more, which had no difference as compared to those age less than 20 years, this might be because as age increases the risk of exposure to HBV increases in parallel, the absence of association in those more than 45 years could be because of the smaller number of individual in this age group as compared to others, only 509 were in the age group as compared to in the age group 20–24 years, 1856.

The prevalence rate of HCV infection is low (0.2%) as compared to previous studies (21, 22, 25) this might be because the study population where most them were volunteer blood donors who were confident of their sero status.

In conclusion, this study had shown that the prevalence rate of HBV, HCV and HIV infections as evidence with positive serological test for HBsAG, anti HCV antibody and anti HIV 1 antibody among blood donors in JUSH was lower compared to previous studies. The study also found no association between HBV or HCV infection with HIV. Thus, community based study should be conducted to ascertain the relationship of HBV or HCV infection with HIV.
